# Modelling the impact of curtailing antibiotic usage in food animals on antibiotic resistance in humans

**DOI:** 10.1098/rsos.161067

**Published:** 2017-04-05

**Authors:** B. A. D. van Bunnik, M. E. J. Woolhouse

**Affiliations:** Centre for Immunity, Infection and Evolution and Usher Institute of Population Health Sciences and Informatics, Ashworth Laboratories, University of Edinburgh, Kings Buildings, Charlotte Auerbach Road, Edinburgh EH9 3FL, UK

**Keywords:** one health, antimicrobial resistance, antibiotic usage, mathematical model, food animals, agriculture

## Abstract

Consumption of antibiotics in food animals is increasing worldwide and is approaching, if not already surpassing, the volume consumed by humans. It is often suggested that reducing the volume of antibiotics consumed by food animals could have public health benefits. Although this notion is widely regarded as intuitively obvious there is a lack of robust, quantitative evidence to either support or contradict the suggestion. As a first step towards addressing this knowledge gap, we develop a simple mathematical model for exploring the generic relationship between antibiotic consumption by food animals and levels of resistant bacterial infections in humans. We investigate the impact of restricting antibiotic consumption by animals and identify which model parameters most strongly determine that impact. Our results suggest that, for a wide range of scenarios, curtailing the volume of antibiotics consumed by food animals has, as a stand-alone measure, little impact on the level of resistance in humans. We also find that reducing the rate of transmission of resistance from animals to humans may be more effective than an equivalent reduction in the consumption of antibiotics in food animals. Moreover, the response to any intervention is strongly determined by the rate of transmission from humans to animals, an aspect which is rarely considered.

## 1. Introduction

Heightened concern about increasing levels of antimicrobial resistance worldwide has led to renewed calls to reduce substantially the use of antibiotics in food animals [1]. The notion that such a reduction could have public health benefits arises primarily from the observation that the volume of antibiotics consumed by food animals worldwide is approaching, and may have already overtaken, the volume consumed by humans [2]. This situation is expected to worsen as the transition to intensive animal production systems continues in many regions, especially China, India, Brazil and other Asian countries [3,4].

Antibiotic consumption by food animals occurs for the purposes of herd health, prophylaxis and growth promotion. Growth promotion, often involving sub-therapeutic doses, is particularly controversial. It has been banned in European Union (EU) countries since 2005 and is the subject of a more recent voluntary ban in the USA. Currently, 51% of the OIE member countries who report on antibiotic consumption/usage have a complete ban on using antimicrobials as growth promoters, and a further 19% have a partial ban [5,6]. There has been some impact on consumption of antibiotics by food animals [7,8] and levels of antibiotic resistance therein [8–10]; however, any consequent benefits to human health are not easily discerned.

A key challenge for understanding the expected impact of reducing drug usage is that the relationship between antibiotic consumption by food animals and levels of resistant bacteria in humans is complex. First, food animals are far from the only source of human exposure to antibiotic resistant bacteria: high levels of antibiotic use in hospitals, clinics and the general population are also major drivers of resistance in humans [11]. Quantifying the specific contribution of the food animal route is not straightforward and has yet to be attempted. Second, there will be many different answers to the quantification question: there are numerous combinations of different antibiotics, bacterial strains and farm animal species, each with their own dynamics, and these are likely to vary between different countries with different healthcare systems and agricultural production systems.

As a first step towards addressing this knowledge gap, here we develop a simple mathematical model for exploring the generic relationship between antibiotic consumption by food animals and levels of resistant bacteria in humans. Our objective is to better understand the dynamics of antimicrobial resistance moving between food animal and human populations and to identify which model parameters have the greatest influence on levels of resistance in humans and for which parameter combinations we expect to see the greatest impact of reducing antibiotic consumption by food animals.

By using the simplest possible mathematical model as a starting point, we hope to be able to make a first step in understanding this highly complex system and gain some robust and useful insights into its behaviour.

## 2. Material and methods

### 2.1. Mathematical model

Our mathematical model is intended to be as simple as possible while still capturing the nonlinearities inherent in infectious agents spreading between two host populations. To achieve this, we consider two variables: *R*_H_, the fraction of humans with antibiotic resistant bacteria (so a measure of the ‘level of resistance’); and *R*_A_, the fraction of food animals with antibiotic resistant bacteria. The rationale of the model is that humans or food animals can acquire antibiotic resistant bacteria from different sources:

(1) within host selection for resistant bacteria in response to direct exposure to antibiotics;
(2) direct or indirect exposure to antibiotic resistant bacteria or mobile genetic elements containing resistance determinants carried by other individuals within the same population (e.g. humans acquiring resistance from a human source); and
(3) as (2) but between different populations (e.g. food animals acquiring resistance from a human source).

The dynamics of *R*_H_ and *R*_A_ is given by the coupled ordinary differential equations:

2.1 $$\frac{\text{d}R_{H}}{\text{d}t}=\Lambda_{H}(1-R_{H})+\beta_{\mathrm{HH}}R_{H}(1-R_{H})+\beta_{\mathrm{AH}}R_{A}(1-R_{H})-\mu_{H}R_{H};$$

$$\frac{\text{d}R_{A}}{\text{d}t}=\Lambda_{A}(1-R_{A})+\beta_{\mathrm{AA}}R_{A}(1-R_{A})+\beta_{\mathrm{HA}}R_{H}(1-R_{A})-\mu_{A}R_{A},$$

where *Λ*_H_ is the *per capita* rate at which humans acquire antibiotic resistant bacteria through direct exposure to antibiotics and *Λ*_A_ is the equivalent in food animals; (1 − *R*_H_) and (1 − *R*_A_) are respectively the fraction of humans without antibiotic resistance and the fraction of food animals without antibiotic resistance. *β*_HH_ is the *per capita* rate at which humans acquire antibiotic resistant bacteria as a result of exposure (directly or indirectly via environmental contamination) to other humans harbouring resistant bacteria and *β*_AA_ is the equivalent for animals; *β*_AH_ is the *per capita* rate at which humans acquire antibiotic resistant bacteria as a result of exposure (directly or, more frequently, indirectly via food products or environmental contamination) to food animals harbouring resistant bacteria and *β*_HA_ is the reverse; *µ*_H_ is the *per capita* rate at which humans with resistant bacteria revert to having only susceptible bacteria (as a combination of clearance of resistance bacterial infections and demographic replacement) and *µ*_A_ is the equivalent in food animals. The time unit is arbitrary and does not affect the equilibrium values. We assume that all transmission parameters (e.g. all *β*'s) combine transmission of both antibiotic resistant bacteria and transmission of mobile genetic elements. Although these rates could be disaggregated, this would (for our study) have no impact on the system dynamics for a given set of parameters.

Studying the system in a steady state (i.e. at equilibrium) allows us to explore the long-term effects of changing different parameter values. To obtain an equation for the equilibrium value, $R_{H}^{*}$, equation (2.1) was set to 0 and solved for *R*_H_ in terms of the eight model parameters. Although we do not consider the real-world system to be at equilibrium, largely because antibiotic consumption patterns have changed considerably in recent decades (from zero before 1932) and continue to do so, we regard $R_{H}^{*}$ as a useful indication of where the model system is tending, and the approach to $R_{H}^{*}$ will be relatively rapid if *µ*_H_ and *µ*_A_ are high, i.e. the mean duration of resistant infections is short (≪1 year).

As an indication of the potential impact of curtailing antibiotic usage in farm animals on the level of resistance in the human population we define $RC_{H}^{*}$ as an adjusted $R_{H}^{*}$ in which *Λ*_A_ = 0 (i.e. no antibiotic usage in food animals) and define impact, *ω*, as 1—the ratio of $RC_{H}^{*}$ to $R_{H}^{*}$:

2.2 $$\omega =1-\frac{{RC}_{H}^{*}}{R_{H}^{*}}.$$

All analyses were carried out in Wolfram Mathematica v. 10.3 [12].

### 2.2. Scenarios

To identify key parameters and capture the nonlinearities of the system in more detail, we consider two distinct scenarios in this study, a low impact scenario and a high impact scenario. The difference between these two scenarios is the choice of baseline value for *β*_HA_. For the low impact scenario, *β*_HA_ = 0.1, and for the high impact scenario, *β*_HA_ = 0.001. These values were chosen to maximize the differences while minimizing changes in the baseline levels of resistance between the two scenarios. The baseline parameters for the two scenarios are given in table 1. The baseline parameter values were chosen such that the long-term prevalence of the fraction of the human population that is affected by resistant bacteria is roughly 70%. This is, for example, consistent with the situation of bacterial resistance to ampicillin in the United Kingdom, where both humans and food animals show similarly high level of resistance [13,14].

**Table 1.** Baseline parameters for the two scenarios.

		value used	
parameter	description	low impact	high impact
*β*_AA_	*per capita* rate at which animals acquire antibiotic resistant bacteria as a result of exposure to other animals harbouring resistant bacteria	0.1	0.1
*β*_HH_	*per capita* rate at which humans acquire antibiotic resistant bacteria as a result of exposure to other humans harbouring resistant bacteria	0.1	0.1
*β*_AH_	*per capita* rate at which humans acquire antibiotic resistant bacteria as a result of exposure to food animals carrying resistant bacteria	0.1	0.1
*β*_HA_	*per capita* rate at which food animals acquire antibiotic resistant bacteria as a result of exposure to humans carrying resistant bacteria	0.1	0.001
*Λ*_H_	*per capita* rate at which humans acquire antibiotic resistant bacteria as a result of direct exposure to antibiotics	0.1	0.1
*Λ*_A_	*per capita* rate at which food animals acquire antibiotic resistant bacteria as a result of direct exposure to antibiotics	0.1	0.1
*µ*_A_	*per capita* rate at which humans with resistant bacteria revert to having only susceptible bacteria	0.1	0.1
*µ*_H_	*per capita* rate at which food animals with resistant bacteria revert to having only susceptible bacteria	0.1	0.1

### 2.3. Sensitivity analysis

We determine which model parameters have most influence on the outcome value by computing the total sensitivity index *D*_Ti_ using the extension of Fourier amplitude sensitivity test (FAST) as described in Saltelli *et al.* [15]. The extended FAST method is a variance-based, global sensitivity analysis technique that has been largely used for studying complex agricultural, ecological and chemical systems (see [16,17] for examples). Independently of any assumption about the model structure (such as linearity, monotonicity and additivity of the relationship between input factors and model output), the extended FAST method quantifies the sensitivity of the model output with respect to variations in each input parameter by means of spectral analysis. It provides measures of the amount of variance of the prevalence that arise from variations of a given parameter in what is called a total sensitivity index, *D*_Ti_. It therefore captures the overall effect of parameter variations on equilibrium levels of resistance over a pre-specified range (i.e. including first- and higher-order interactions between model parameters). For example, a value of *D*_Ti_ = 0.10 indicates that 10% of the total observed variation of the prevalence is explained by the parameter under consideration. The sensitivity analysis was carried out using R [18]. For the sensitivity analysis, we used a parameter range of 0.0 to 1.0 for all parameters under investigation.

## 3. Results

**Figure 1.**
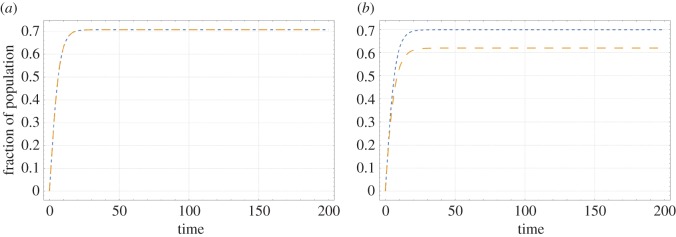
Trajectory of the fraction of the human population with antibiotic resistant bacteria (*R*_H_) and the fraction of food animals with antibiotic resistant bacteria (*R*_A_) in time for the low impact scenario (*a*) and the high impact scenario (*b*). Blue curves represent *R*_H_, orange curves represent *R*_A_.

For the two scenarios considered here, figure 1 shows the trajectory of *R*_H_ and *R*_A_ in time. Figure 1 shows that the long-term prevalence of resistance in the human population stabilizes to a value of 0.71 for the low impact scenario and 0.70 for the high impact scenario. The long-term prevalence of resistance in the animal population stabilizes to a value of 0.71 for the low impact scenario and 0.62 for the high impact scenario. These differences between the scenarios are because of the lower value of *β*_HA_ in the high impact scenario (table 1). These prevalences are consistent with scenarios encountered in practice (see Material and methods).

**Figure 2.**
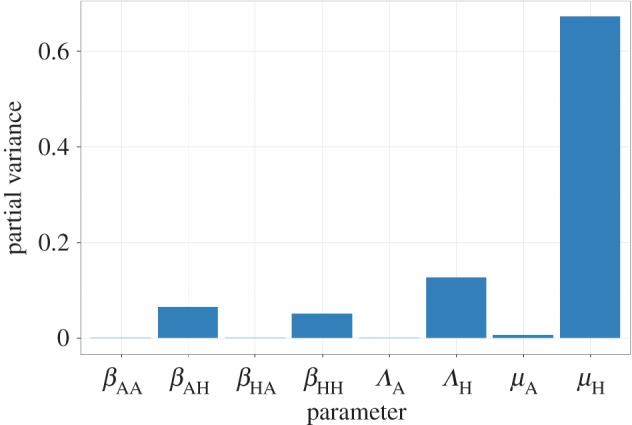
Results of a global sensitivity analysis on the equilibrium equation showing the partial variance of the individual model parameters. Higher bars indicate greater sensitivity of the model to that parameter. See Material and methods section for details about the sensitivity analysis and parameter ranges used.

The sensitivity analysis on the equilibrium equation for $R_{H}^{*}$ shows that this equilibrium is most sensitive to changes in *µ*_H_, followed by *Λ*_H_, *β*_AH_ and *β*_HH_, (figure 2). Furthermore, the system is minimally sensitive to changes in ‘animal’ parameters (*β*_AA_, *β*_HA_, *Λ*_A_ and *µ*_A_).

**Figure 3.**
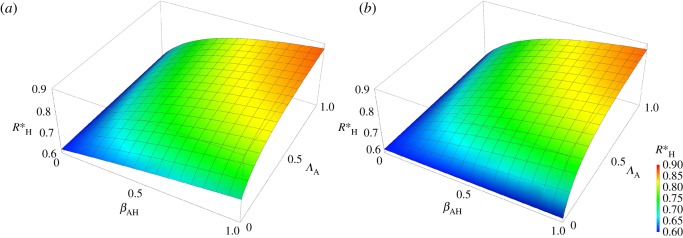
Surface of $R_{H}^{*}$ as a function of *β*_AH_ and *Λ*_A_ for the low impact scenario (*a*) and the high impact scenario (*b*).

Figure 3 shows that, in accordance with the sensitivity analysis, the equilibrium $R_{H}^{*}$ is relatively insensitive to changes in *Λ*_A_, but is more sensitive to changes in *β*_AH_, suggesting that reducing the former without addressing the latter may have limited impact on the prevalence of antibiotic resistance in the human population. Furthermore, the figure shows that the change in $R_{H}^{*}$ owing to *β*_AH_ is nonlinear, which suggests that partial reductions of *β*_AH_ may only have limited impact when $R_{H}^{*}$ is already high. Comparing figure 3*a* with figure 3*b* shows that the effect of reducing the rate at which animals acquire antibiotic resistant bacteria as a result of exposure of animals to antibiotics (*Λ*_A_) is also strongly influenced by the *per capita* rate at which food animals acquire antibiotic resistant bacteria as a result of exposure to humans carrying resistant bacteria (*β*_HA_). For the higher value of *β*_HA_ (low impact scenario, figure 3*b*) lowering *Λ*_A_ by itself has little influence.

**Figure 4.**
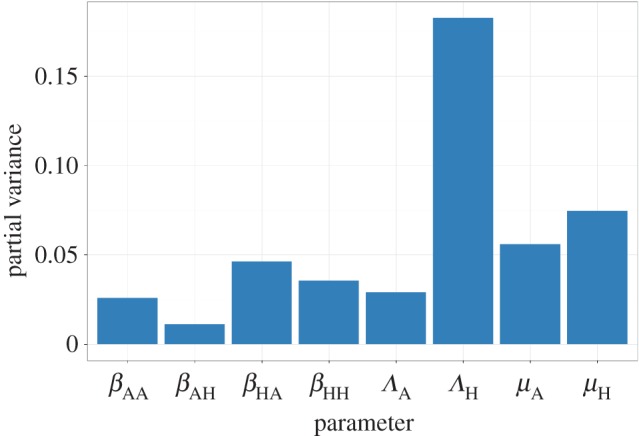
Results of a global sensitivity analysis on the impact (*ω*, defined in the Material and methods section) showing the partial variance of the individual model parameters. Higher bars indicate greater sensitivity of the model to that parameter. See Material and methods section for details about the sensitivity analysis and parameter ranges used.

**Figure 5.**
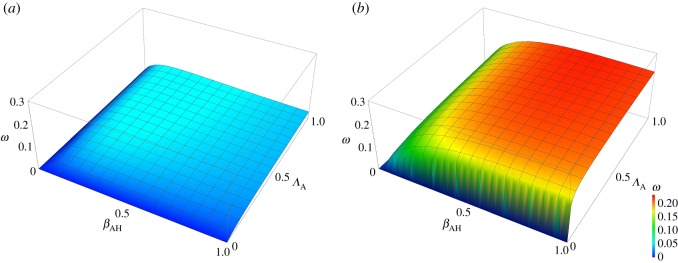
Surface of impact, *ω*, as a function of *β*_AH_ and *Λ*_A_ for the low impact scenario (*a*) and the high impact scenario (*b*).

Figures 4 and 5 show the results for the impact of curtailing antibiotic use in food animals, quantified as the variable *ω* (defined above). Figure 4 shows the sensitivity of the impact to the different parameters. From figure 4, it is clear that the impact is most sensitive to *Λ*_H_, the rate at which humans acquire antibiotic resistant bacteria as a result of exposure of humans to antibiotics, followed by *μ*_H_, *μ*_A_ and *β*_HA_. *Λ*_A_ and *β*_AH_ have less influence. Figure 5 shows the effects of varying *β*_AH_ and *Λ*_A_ on the impact, *ω*, of curtailing antibiotic use in food animals. The difference between the low impact scenario and the high impact scenario is immediately clear from these graphs as there is virtually no impact of reducing *Λ*_A_ in the low impact scenario (with relatively high *per capita* rate of transmission from humans to food animals, *β*_HA_), while in the high impact scenario (with relatively low *β*_HA_) there is a much more obvious benefit.

## 4. Discussion

It is often implied that the high levels of consumption of medically important antibiotics by food animals is contributing significantly towards the global public health problem of antibiotic resistance. Therefore, we tested the potential impact of curtailing the use of antibiotics in food animals on the (long-term) prevalence of humans carrying resistant bacteria using a mathematical model designed to capture the nonlinearities inherent in the transmission of infectious agents between two populations as simply as possible. Our results show that, as expected, the system is sensitive to changes in *per capita* rate at which humans acquire antibiotic resistant bacteria as a result of direct exposure to antibiotics (*Λ*_H_). Of much greater interest is the importance of the *per capita* rate of transmission of antimicrobial resistance from humans to animals (*β*_HA_) (figures 4 and 5). For this reason, we compared two scenarios, a low impact scenario (high *β*_HA_) and a high impact scenario (low *β*_HA_). If *β*_HA_ is high (figures 3*a* and 5*a*) then the effects of reducing the rates at which animals acquire resistance as a result of antibiotic usage (*Λ*_A_) and humans acquire antibiotic resistant bacteria from animals (*β*_AH_) are limited (figures 3*a* and 5*a*, when *β*_AH_ or *Λ*_A_ approaches 0). This contrasts with the situation where *β*_HA_ is low (figures 3*b* and 5*b*). This indicates that whenever the rate of transmission of antibiotic resistant bacteria from humans to animals is high it is more difficult to curb the antibiotic resistance problem, a rather counterintuitive result and often overlooked in discussion about this topic.

Also of interest is that a failure to address the agricultural usage of antibiotics severely limits what can be achieved by tackling the problem from the human side, i.e. even if no resistance is acquired via direct exposure to antibiotics in humans (achieved by reducing *Λ*_H_ to 0), we can only reduce the long-term prevalence of antibiotic resistance in humans to 0.56 for the low impact scenario and 0.54 for the high impact scenario (see the electronic supplementary material, figure S1). In other words, if resistance dynamics in human and animal populations are coupled, as is generally thought to be the case in practice, substantial impacts on levels of resistance requires coordinated interventions across both populations.

Our study has several limitations that should be recognized. The first limitation is the simplicity of the model used. As indicated in the introduction, antibiotic resistance is a highly complex problem with numerous routes of transmission and with dynamics that will vary qualitatively and quantitatively for different drug-bug-animal combinations. Accounting for all these different routes and combinations separately is challenging. However, by taking the simplest possible mathematical model as a starting point, we are able to make a first step in trying to understand this highly complex system and gain some robust and useful insights into its behaviour. These findings can then be used as stepping stones for the development of more complicated (and perhaps more precise) models. As with all models, several assumptions have been made in this study. For example, *Λ* is clearly related to antibiotic consumption, but the shape of this relationship is left undefined in this study as we are only interested here in the specific alternative scenario, where *Λ*_A_ = 0. For a partial reduction in *Λ*_A_, however, this relationship would need to be specified as different relationships could lead to (very) different results. Similarly, for the *β*'s we combined the rate of acquiring resistance through transmission of (clonal) bacteria and through the transmission of mobile genetic elements. Although it is possible to disaggregate these modes of transmission, this would require an additional parameter and would not alter the system dynamics for a given set of parameters (and thus would not alter our results). However, if the separate contributions of the different transmission paths were of interest, such disaggregation would be necessary. Lastly, for the recovery rates (*µ*) we assumed that these combine recovery from infection with a resistant strain with demographic replacement by hosts with susceptible ones. The scenarios considered here assume high recovery rates and so emphasize the former (given that human and food animal demographies are very different).

Key aspects of future models might include: (i) information on geographical distance and contact structures between and within populations; (ii) explicit consideration different modes of inheritance of resistance; and (iii) quantitatively relating rates of gain of resistance to historical data on antibiotic consumption. Also, the dependency of resistance on demographics should be taken into account as human and livestock demographics can be very different (for example the batch structure that is common in poultry has an influence on infectious disease [19]).

As with all models, parametrization is an important issue. In this study, we chose our baseline parameter values such that the long-term prevalence of the fraction of the human population that is affected by resistant bacteria is roughly 70%. This mimics, for example, the situation of ampicillin resistance in both human and livestock in the United Kingdom where antibiotic resistance is well established but still leaves room for improvement or deterioration of the levels of resistance. However, knowledge of the prevalence of resistance in the two populations is not by itself sufficient to parametrize this model, as there are eight parameters to be estimated. Independent estimates of parameter values are required, but are not currently available, even for any specific drug-bug-animal combination in a defined setting. In practice, the many different combinations of antibiotic, bacteria strain, food animal species and setting will represent many different points in parameter space, each of which would need to be determined individually—a significant challenge. That said, the results of the simple, generic model presented here are robust in the sense that they apply over a wide range of parameter space that we expect to cover many real-world scenarios.

## 5. Conclusion

To conclude, we have shown in this study that we can obtain useful insights into a highly complex problem like antibiotic resistance by using a simple mathematical model. Although it is widely regarded as intuitively obvious that reducing antibiotic consumption in animals would decrease levels of antibiotic resistance in humans this is, in fact, not the case for a wide range of scenarios (i.e. parameter space), especially if this intervention is made in isolation. Reducing the rate of transmission of resistance from animals to humans may often be more effective. In addition, the behaviour of the system, and so the response to any intervention, is strongly determined by the rate of transmission from humans to animals, but this has received almost no attention in the literature. It is thus not enough to only lower the consumption of antibiotics in food animals, the transmission both from and to food animals should also be limited in order to maximize the impact of this and other interventions. We recommend that formal, quantitative analyses are needed to assess the expected benefits to human health of reducing antibiotic consumption by food animals. In some circumstances, these benefits will be very small and other measures will be needed to reduce the public health burden of antibiotic resistance.

## Supplementary Material

Supplementary Information

## Data accessibility

All code and data supporting the results in this article is made publicly available via Dryad: http://dx.doi.org/10.5061/dryad.1g98m [20].
