# Supplementary material for: Modelling the impact of curtailing antibiotic usage in food animals on antibiotic resistance in humans
Source: R Soc Open Sci. 2017 Apr 5;4(4):161067. doi: 10.1098/rsos.161067 (PMC5414261; doi:10.1098/rsos.161067)
Supplement: Supplementary Information [file rsos161067supp1.docx]

# Supplementary Information


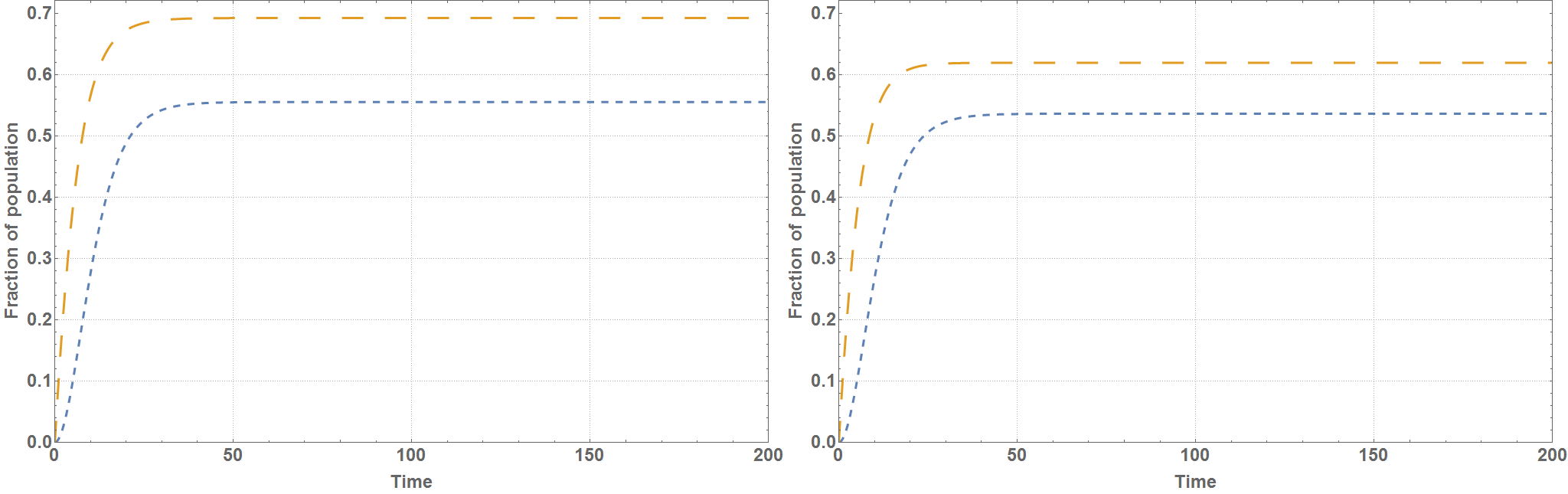


Figure S1. Trajectory of the fraction of the human population with antibiotic resistant bacteria (*R_H_*) and the fraction of food animals with antibiotic resistant bacteria (*R_A_*) in time for the low impact scenario (panel A) and the high impact scenario (panel B), using *Λ_H_*=0, showing the effect of curtailing antibiotic usage in humans. Blue curves represent *R_H_*, orange curves represent *R_A_*.
